# Molecular Mechanisms and Applications of Polyphenol-Protein Complexes with Antioxidant Properties: A Review

**DOI:** 10.3390/antiox12081577

**Published:** 2023-08-08

**Authors:** Yulin Feng, Chengming Jin, Shihao Lv, Huijuan Zhang, Feiyue Ren, Jing Wang

**Affiliations:** China-Canada Joint Lab of Food Nutrition and Health (Beijing), Key Laboratory of Special Food Supervision Technology for State Market Regulation, School of Food and Health, Beijing Technology & Business University (BTBU), Beijing 100048, China; 2150021001@st.btbu.edu.cn (Y.F.); 2130021004@st.btbu.edu.cn (C.J.); 2130022045@st.btbu.edu.cn (S.L.); 20200101@btbu.edu.cn (F.R.)

**Keywords:** complex, interaction, antioxidation, free radical scavenging, lipid peroxidation, delivery system

## Abstract

Proteins have been extensively studied for their outstanding functional properties, while polyphenols have been shown to possess biological activities such as antioxidant properties. There is increasing clarity about the enhanced functional properties as well as the potential application prospects for the polyphenol–protein complexes with antioxidant properties. It is both a means of protein modification to provide enhanced antioxidant capacity and a way to deliver or protect polyphenols from degradation. This review shows that polyphenol–protein complexes could be formed via non-covalent or covalent interactions. The methods to assess the complex’s antioxidant capacity, including scavenging free radicals and preventing lipid peroxidation, are summarized. The combination mode, the type of protein or polyphenol, and the external conditions will be the factors affecting the antioxidant properties of the complexes. There are several food systems that can benefit from the enhanced antioxidant properties of polyphenol–protein complexes, including emulsions, gels, packaging films, and bioactive substance delivery systems. Further validation of the cellular and in vivo safety of the complexes and further expansion of the types and sources of proteins and polyphenols for forming complexes are urgently needed to be addressed. The review will provide effective information for expanding applications of proteins and polyphenols in the food industry.

## 1. Introduction

**Figure 1:**
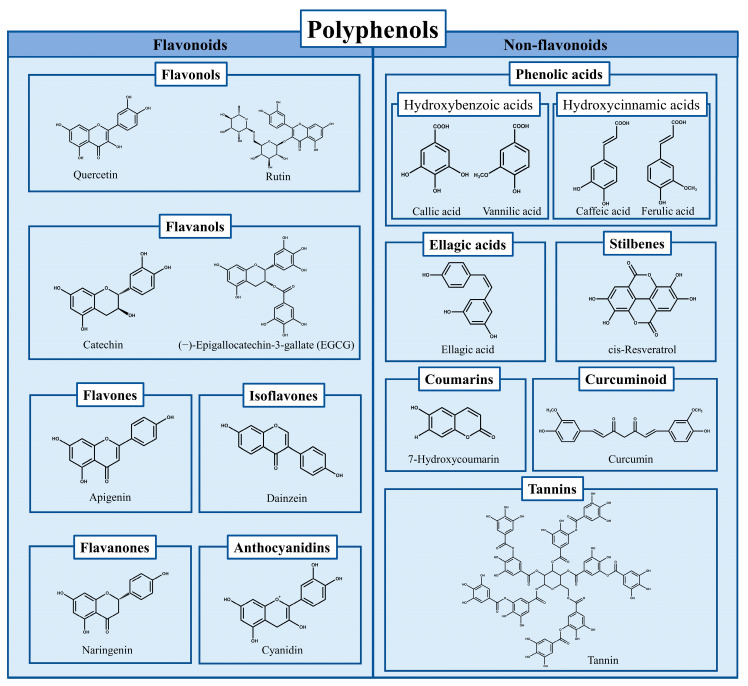
The classification of polyphenols and the chemical formulas of representative compounds.

Protein’s excellent functional properties make it an important ingredient of food engineering a foaming agent, emulsion, film, gel, etc. [1]. It is also focused on improving its functional characteristics through appropriate structural modification [2]. Polyphenols (including flavonoids, phenolic acids, tannins, stilbene, curcumin, Figure 1) have excellent biological activity such as antioxidant capacity and are therefore expected to be more widely used in food systems. However, the low stability, degradability, and susceptibility to oxidation limit their application [3]. In recent years, the complexes formed by proteins (such as whey protein, egg protein, soy protein, zein, gelatin, casein) combined with polyphenols (including flavonoids, phenolic acids, tannins, stilbene, lignin, curcumin) has become both an effective mean of protein modification or processing with expanded functions and a way to protect or deliver polyphenols with biological activities from degradation, therefore, have shown increased attention and research heat [4,5,6,7,8,9,10].

Polyphenols can bind to proteins either covalently or non-covalently [5,7]. The enhanced antioxidant activity and functional properties of the complexes, such as emulsifying, gelling, and stability, are being widely studied [7]. Among them, antioxidant activity is one of the most essential characteristics of polyphenol–protein complexes. On the one hand, combining the proteins and polyphenols can introduce the active hydroxyl group from polyphenols into proteins so that it can endow proteins with significantly increased antioxidant activity [11,12,13]. This makes the polyphenol–protein complexes used as antioxidant emulsifiers, antioxidant films, etc. [7]. The emulsion prepared by Ren et al. [14] using the covalent combination of zein and resveratrol had higher antioxidant activity. The food packaging films prepared by Jiang et al. [15] applying the interaction between proteins and polyphenols showed high free radical scavenging activities. On the other hand, the combination with proteins can protect polyphenols from degradation and has better antioxidant stability [3]. Zou et al. [16] reported that the antioxidant activity of grape seed procyanidins could be protected from reducing activity loss during storage by soy protein isolates. The resveratrol encapsulated with nano-delivery particles constructed by Fan et al. [17] by conjugating bovine serum albumin (BSA) and caffeic acid had better digestive stability and cell antioxidant capacity. Furthermore, polyphenols and proteins can sometimes work synergistically to counteract oxidative [18]. Although there are some reviews on this aspect, a comprehensive summary of antioxidant properties as one of the most important functional properties of the complexes is still lacking.

This article reviewed the preparation path of the complexes and the interaction mechanism between polyphenols and proteins, including covalent and non-covalent binding. The assessment methods and the influencing factors of antioxidant capacity were summarized, the potential applications of the complexes in food engineering were reviewed, and the inadequate aspects and perspectives were indicated. A comprehensive understanding of the polyphenol–protein complexes with antioxidant properties is essential to promote them to be effective means to improve the functional properties and biological activity of proteins and polyphenols and expand their application in the food field.

## 2. Interaction Mechanism and Preparation of Polyphenol–Protein Complexes

**Figure 2:**
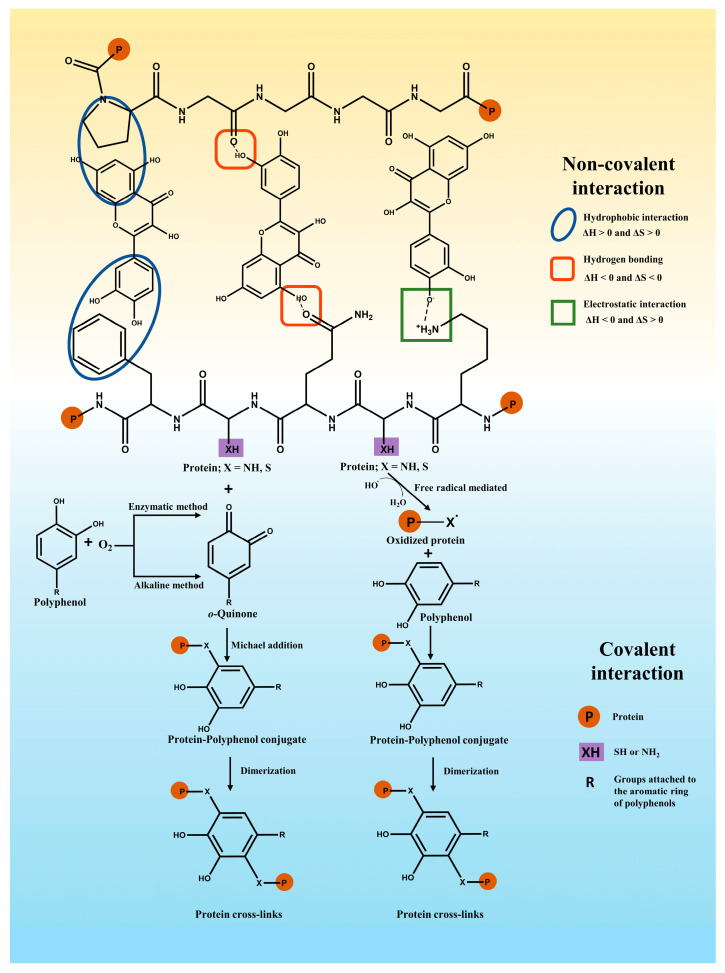
Major non-covalent interaction forces (the **top** half) between polyphenols and proteins and covalent bonding of polyphenols to proteins (the **bottom** half).

In general, polyphenols may bind with proteins via covalent or non-covalent interactions [19] (Figure 2). An example of non-covalent interaction is hydrogen bonding, hydrophobic forces, electrostatic interactions, and van der Waals forces. In comparison, the covalent binding of polyphenols and proteins can be obtained primarily by enzymatic, alkaline, radical grafting, or chemical coupling methods [20].

### 2.1. Non-Covalent Interactions

Non-covalent interactions between proteins and polyphenols may typically include hydrophobic interactions, hydrogen bonds, electrostatic interactions, and van der Waals interactions, which are reversible and weaker than covalent interactions [3,21].

In general, the formation of polyphenol–protein complexes relies mainly on hydrogen bonding and hydrophobic interactions, followed by other interactions (e.g., electrostatic interactions) [7]. When proteins and polyphenols interact with each other, alterations in the total strength of molecular interactions cause changes in the heat of the system. Information about the combined thermodynamic parameters (ΔH, ΔS, and ΔG) has been used to determine the nature of the interaction forces involved [22]. The main non-covalent interactions for ΔH > 0 and ΔS > 0 involve hydrophobic interactions. The main non-covalent interactions for ΔH < 0 and ΔS < 0 involve hydrogen bonding and van der Waals interactions. ΔH < 0 and ΔS > 0 interactions have been mainly attributed to electrostatic interactions [5,22].

Hydrogen bonding interactions are one of the main drivers of polyphenols binding to proteins. Hydrogen bonding is the interaction involving a hydrogen atom located between a pair of other atoms having a high affinity for electrons. As for polyphenols, they act as hydrogen donors, and their hydroxyl groups can form hydrogen bonds through interactions between the C=O groups of the amide group on the peptide chain, the oxygen or nitrogen on the side chains of amino acid residues, especially hydroxyl (–OH) and amino (–NH2) groups [23,24]. Zhang et al. [25] found that ferulic acid, quercetin, and vanillic acid could interact via three, seven, and two hydrogen bonds with β-lactoglobulin, respectively. Wen et al. [26] revealed that ovalbumin and procyanidin have a hydrogen bond-dominated interaction. Jiang et al. proved that Trp 118, Glu 11, and Lys 5 of α-lactalbumin could form hydrogen bonds with hydroxy safflower yellow A, respectively [11]. 

In addition to hydrogen bonding, hydrophobic interactions are one of the main driving forces of polyphenol–protein binding [3,6]. Hydrophobic interaction is usually understood as the force that the hydrophobic groups cluster together to avoid contact with water. The hydrophobic interactions rely on the fact that the non-polar aromatic ring in the phenolic compounds interacts hydrophobically with the hydrophobic amino acid residues of proteins (alanine, cysteine, glycine, isoleucine, leucine, methionine, phenylalanine, tyrosine, tryptophan, and valine) [5,27]. For instance, the aromatic ring and aliphatic chain of curcumin could interact hydrophobically with the hydrophobic region (residues 971-1410) of myosin [28]. Rosmarinic acid was inserted into the hydrophobic pocket formed by the amino acid residues Ser191, Arg198, Leu237, His241, Leu259, Ile263, His287, Ala290, and Glu291 of BSA and bound to the hydrophobic amino acids in the lumen of BSA through hydrophobic interactions [29]. The catechol part of chlorogenic acid interacted via the PI-PI accumulation of hydrophobic force with β-lactoglobulin Phe105 [30].

Electrostatic interactions occur as an attraction force that is created between two completely or partially ionized species with opposite charges. Electrostatic interactions between proteins and phenolics usually involve the deprotonation of some phenolic acids with low pK_a_ values (e.g., cinnamic acid derivatives such as ferulic acid) under neutral conditions. At this point, positively charged protein groups, such as the ε-amino group of lysine, would react with the hydroxyl groups with a high electronegativity of the polyphenol [3,23]. For instance, ferulic acid has been found to interact electrostatically with sites containing positively charged amino acid residues in BSA since ferulic acid (pK_a_ = 4.58) is a negatively charged molecule at pH 7.4 [31]. Similarly, the electrostatic interactions are more important for the binding of caffeic acid, ferulic acid, and chlorogenic acid (pK_a_ = 3.45, 3.58, and 3.33, respectively) to β-casein since they are more readily ionized in aqueous solution under physiological pH conditions [32]. 

Van der Waals forces relatively attract the weak forces between molecules arising from electron fluctuations and the interaction of dipole moments. Van der Waals forces are usually generated in combination with other interactions [3,7]. Rosmarinic acids interacted with β-lactoglobulin or α-lactalbumin with the driving forces of hydrogen bonds, hydrophobic forces, and van der Waals force [29]. The binding of ferulic acid/quercetin/vanillic acid to β-lactoglobulin involved various non-covalent interactions such as hydrogen bonds, van der Waals interactions, and hydrophobic interactions [25]. Ovalbumin-isoquercitrin complexes are formed by hydrophobic interactions, van der Waals forces, and hydrogen bonding [33].

### 2.2. Covalent Interactions

When covalent binding between polyphenols and proteins occurs, it is an irreversible interaction because of the chemical reactions involved [34]. The process mainly concerns the oxidation of polyphenols to strongly electrophilic quinones. Then, the interaction of the quinones with nucleophilic amino acid (cysteine, lysine, methionine, and tryptophan) residues on proteins or peptides via Michael addition forms covalent cross-linking [35]. Quinones can interact with sulfhydryl, amino, guanidinium, or imidazole groups on proteins or peptides [35], where free sulfhydryl groups have been identified as being more susceptible to covalent cross-linking than other groups [36].

Enzymatic and non-enzymatic methods are applied to mediate the covalent binding of polyphenols to proteins [6,37]. Enzymatic methods are environmentally friendly and highly specific methods that synthesize complexes with intense free radical scavenging activity. However, the preparation procedures are complex and expensive [38]. In this scheme, firstly, phenolase (monophenolase or cresolase) induces the oxidation of polyphenols to o-diphenols. Subsequently, under oxygen conditions, o-diphenolase (laccase or catecholase) converts the o-diphenols to o-quinones, and the active quinones can interact with nucleophilic amino acid residues in the protein chain to form cross-linked proteins or polymers [39,40]. For instance, Temdee and Benjakul [41] used laccase oxidized gallic acid and catechuic acid covalently cross-linked with gelatin from cuttlefish (Sepia pharaonis) skin to improve its gel functional properties. Velickovic and Stanic-Vucinic [39] et al. used tyrosinase and laccase to achieve covalent binding of caffeic acid to β-casein or β-lactoglobulin, and the reduced solubility and in vitro digestibility of the complexes were found.

The alkaline reaction is one of the common non-enzymatic methods for binding polyphenols and proteins. The oxidation of polyphenols leads to the formation of semiquinones, which are rearranged into quinones under alkaline and aerobic conditions. These intermediates can form covalent crosslinks between proteins and polyphenols (C-N or C-S) [7,20,40]. Parolia et al. [18] obtained the conjugate of lentil protein and quercetin/rutin /ellagic acid prepared by the alkaline reaction, and the enhanced antioxidant properties of the conjugates were observed. Xu et al. [42] formed the conjugates by the binding of chlorogenic acid, gallic acid, and caffeic acid with zein under alkaline conditions and investigated the effects of covalent interactions on the structural and functional properties of the proteins. 

For non-enzymatic methods, free radical grafting with ascorbic acid and hydrogen peroxide as radical inducers is considered an effective synthetic method for the preparation of protein–polyphenol complexes with high bioactivity, low cost, and non-toxic chemicals involved [38,43]. The process mainly involves the oxidation of amino acids located on the side chains of proteins by free radical initiators to form free radicals, which then react with polyphenols through covalent bonds to form polyphenol–protein conjugates with strong interactions as well as high stability [44,45]. The wheat gluten hydrolysate-chlorogenic acid conjugate was obtained via the free radical method to explore the potential application in improved functional properties of the conjugates [46]. A camel whey–quercetin conjugate was prepared using the redox pair consisting of ascorbic acid and H_2_O_2_ by Baba et al. [47].

In addition to the above methods, some chemical cross-linking agents were also applied to prepare polyphenol–protein conjugates [20]. The conjugates prepared from zein assembled with chlorogenic acid or gallic acid via the chemical method obtained by Xu et al. [48] had high polyphenol content and grafting efficiency. 1-ethyl-3-(30-dimethylaminopropyl) carbodiimide (EDC) and N-hydroxysuccinimide (NHS) were applied to achieve caffeic acid binding with β-lactoglobulin for the development of adding functionality to milk-based protein [49].

**Figure 3:**
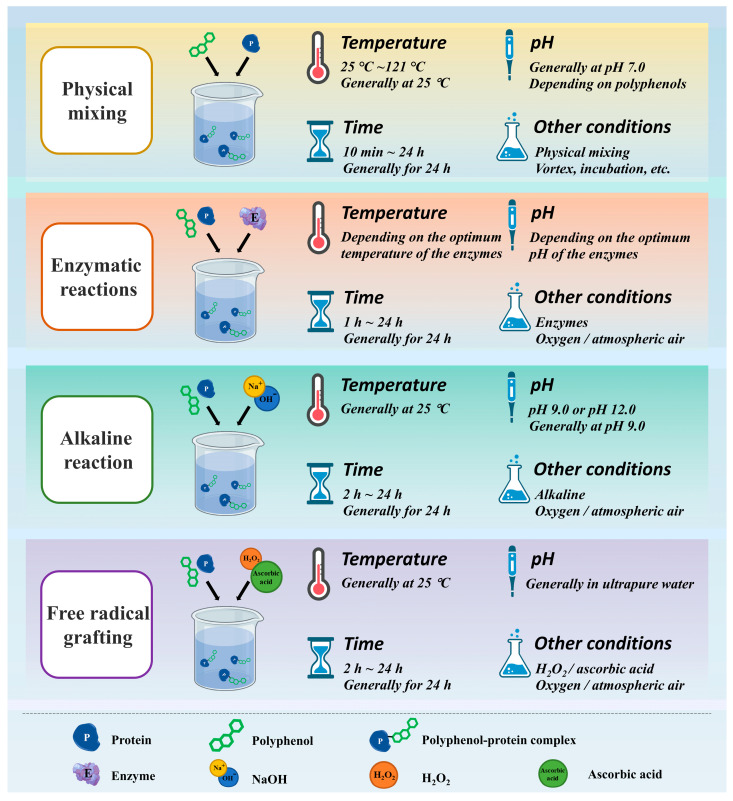
Production paths of protein–polyphenol complexes.

According to the mechanism of interaction between polyphenol and protein presented above, the polyphenol–protein complexes can be prepared by physical mixing (non-covalent interaction), enzyme reaction, alkaline reaction, and free radical grafting (Figure 3). In general, different combination modes will be selected according to the various research purpose or the functional characteristics and biological activities intending to be improved. On the one hand, the preparation of non-covalent polyphenol–protein complexes (i.e., simple physical mixing under appropriate extrinsic conditions) is more conveniently prepared than covalently combined complexes [4]. Since the non-covalent interaction of the complexes is reversible, it is possible to achieve the binding of polyphenols to proteins during preparation and release of polyphenols during digestion [3,37]. Therefore, this combination mode is generally used to explore the digestion or release characteristics of the combined polyphenols, especially the protective effect of protein on antioxidant or other bioactive properties of polyphenols in the process of storage, intake, or digestion [12,30,50]. On the other hand, the covalent interaction between polyphenol–protein complexes is irreversible, which makes the complexes more stable. At the same time, covalent binding can mediate the high grafting rate of polyphenol binding to proteins so that polyphenol–protein complexes with higher antioxidant properties can be obtained [38,42,48]. Therefore, the complexes of covalent graft complexes are mainly used to prepare protein-based emulsifiers, delivery carriers, etc. Notably, the safety and potential hazard of the generated “new complexes” should also be considered, especially the conjugated complexes prepared via the irreversible chemical reaction. Therefore, researchers should consider the investigation purpose and application aspects to select the binding mode of polyphenol–protein complexes.

## 3. Assessment of Antioxidant Properties of Polyphenol–Protein Complexes

**Figure 4:**
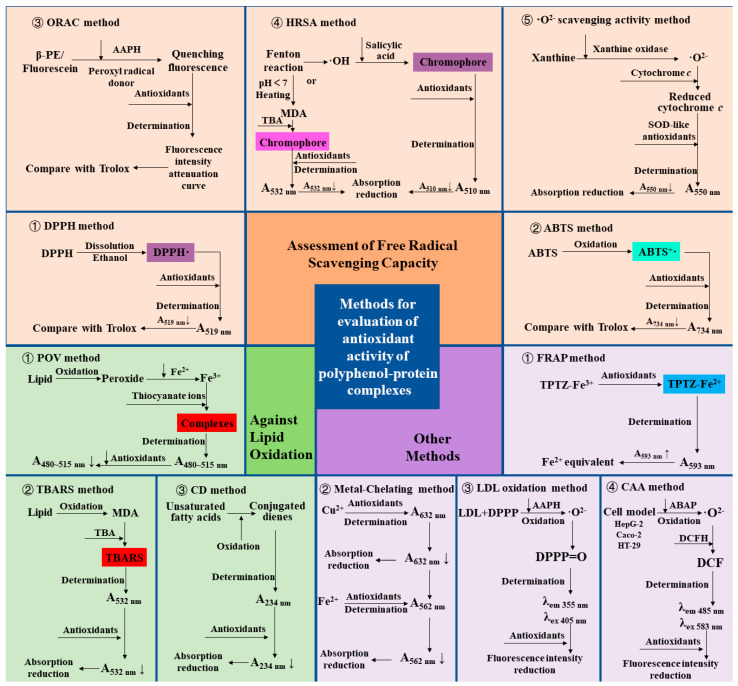
Assessments and principles of Antioxidant Capacity of polyphenol–protein complex. DPPH: 2,2-diphenyl-1-picrylhydrazyl radical; ABTS: 2,2′-azinobis (3-ethylbenzthiazoline-6-sulfonic acid) radical; ORAC: Oxygen radical absorbance capacity; β-PE: β-phycoerythrin; AAPH: 2,2′-Azobis (2-amidopropane) dihydrochloride; HRSA: Hydroxyl radical scavenging activity; MDA: Malondialdehyde; TBA: Thiobarbituric acid; ·O^2−^: Superoxide anion radical; SOD: Superoxide dismutase; FRAP: Ferric ion reducing antioxidant power; TPTZ-Fe^3+^: Tripyridine triazine ferric; TPTZ-Fe^2+^: Tripyridine triazine ferrous; LDL: Low-density lipoprotein; DPPP: Diphenyl-1-pyrenylphosphine; AAPH: 2,2’- azobis (2-amidinopropane) dihydrochloride; DPPP = O: Diphenyl-1-pyrenyl phosphine oxide; CAA: Cellular antioxidant activity; ABAP: 2,2′-azobis(2-amidinopropane) dihydrochloride; DCFH: Dichlorofluorescin probe; DCF: Dichlorofluorescein; POV: Peroxide value; TBARS: Thiobarbituric acid reactive substances; CD: Conjugated dienes.

The methods used to assess antioxidants are abundant, and here is a summary and review of the methods commonly used to evaluate the antioxidant properties of polyphenol–protein complexes. The various assessment methods and their principles are summarized in Figure 4 and Table 1.

### 3.1. Assessment of Free Radical Scavenging Capacity

The method to determine the antioxidant capacity of the complexes by assessment of free radical scavenging capacity is the most popular and used method at present because it is simple and fast. Among them, assessments of 2,2-diphenyl-1-picrylhydrazyl (DPPH) radical and 2,2′-azinobis (3-ethylbenzthiazoline-6-sulfonic acid) (ABTS) radical scavenging capacities are the most widely used methods. DPPH· ethanol solution is purple and has strong ultraviolet absorption at 517 nm. After adding antioxidants, the antioxidant reacts with DPPH·, making the reaction system lighter in color, and the absorbance value decreases [74]. In the ABTS method, ABTS, as the chromogenic agent, produces a stable blue-green cationic radical ABTS+· after oxidation, and then the reaction system is discolored by adding antioxidants. The absorbance was measured at 734 nm, and a decrease in absorbance was observed [75]. The results of the DPPH or ABTS radical scavenging capacity of the antioxidants were mostly expressed in terms of the equivalent concentration of Trolox [76]. It is reported that the combination of chlorogenic acid and the proteins (zein, β-lactoglobulin, wheat gluten hydrolysate) showed enhanced capacities of scavenging DPPH and ABTS free radicals [30,42,46,48]. 

In addition to DPPH and ABTS methods, the oxygen radical absorbance capacity (ORAC) method, hydroxyl radical scavenging activity (HRSA) method, and superoxide anion radical (·O^2−^) scavenging activity method is also used to evaluate the free radical scavenging capacity of polyphenol–protein complexes. 

In the ORAC method, β-phycoerythrin (β-PE) or fluorescein (3′,6′-dihydroxyspiro[isobenzofuran-1[3H], 9′[9H]-xanthen]-3-one) are used as the fluorescent indicator protein, and the 2,2′-Azobis (2-amidopropane) dihydrochloride (AAPH) and Cu^2+^-H_2_O_2_ system are used as the sources of lipid peroxidation free radicals or hydroxyl free radicals, with the Trolox as the reference in general. When β-PE is attacked by free radicals, the fluorescence decreases continuously at a certain wavelength. The free radical scavenging ability of the sample is calculated according to the change in its fluorescence intensity attenuation curve [77,78]. In the HRSA method, one is to generate hydroxyl radicals to initiate the Fenton reaction. The oxidation degree of the system is evaluated by measuring the amount of Fenton reaction products. The other is evaluated by measuring the amount of hydroxyl radicals produced by the Fenton reaction. In the former, deoxyribose, iron, and EDTA produce hydroxyl radicals to induce the Fenton reaction. When heated under acidic conditions, malondialdehyde (MDA) is produced, and thiobarbituric acid (TBA) will interact to form a pink chromophore with absorption at 532 nm. The degree of oxidation of the system is evaluated by measuring the absorbance at 532 nm. In the latter, H_2_O_2_ /Fe^2+^ system generates hydroxyl radicals through the Fenton reaction and produces purple compounds with salicylic acid addition. After adding antioxidants, the reduction of absorption at 510 nm was measured to reflect its hydroxyl radical scavenging ability [79]. In ·O^2−^ scavenging activity method, O^2−^ produced in the xanthine/xanthine oxidase system can reduce a certain amount of oxidized cytochrome c to reduced cytochrome c, which has the maximum light absorption at 550 nm. In the presence of superoxide dismutase (SOD) or SOD-like antioxidants, due to their catalytic disproportionation of a part of O^2−^, the amount of reduced cytochrome c is correspondingly reduced, and the absorbance at 550 nm is reduced so as to evaluate the SOD-like O^2−^ scavenging activity of antioxidants [65].

Fan et al. [57] prepared whey protein isolate-(−)-epigallocatechin-3-gallate (EGCG) conjugate by free radical grafting and used it as an emulsifier to stabilize menhaden oil. The results showed that the ORAC of the conjugate was enhanced compared with whey protein isolate alone. The results of the HRSA of Abd El-Maksoud et al. [49] showed that the covalently conjugate formed by β-lactoglobulin and chlorogenic acid has a more robust antioxidant capacity than the non-covalent complex. Chung et al. [65] found that gelatin did not have superoxide anion scavenging activity, while the enzyme synthesized gelatin catechin conjugate gave SOD-like activity.

### 3.2. Other Assessment of Antioxidant Properties

The ferric ion-reducing antioxidant power (FRAP) method is widely used to determine the antioxidant activity indirectly (i.e., the ability of the tested substance to reduce ferric iron to ferrous iron) [80]. Yin et al. [60] found that after β-casein was combined with chlorogenic acid, the FRAP value of the complex was significantly higher than the sum of β-casein alone and chlorogenic acid alone, indicating a synergistic effect. Another report showed that the FRAP values of chlorogenic acid in the presence of β-lactoglobulin were significantly increased at high temperatures (85–121 °C) compared to the values of chlorogenic acid alone [61]. 

The antioxidant activity can also be evaluated by measuring the ability of antioxidants to chelate metal ions, which are one of the essential sources of free radicals [81]. It has been reported that the metal-chelating activity of silk sericin increased after modification by hydroquinone and pyrogallol, thus systematically reducing the degree of oxidation [64]. 

In the low-density lipoprotein (LDL) oxidation method, LDL is labeled by diphenyl-1-pyrenylphosphine (DPPP), a fluorescent probe that can reflect hydrogen peroxide produced by lipid oxidation [65]. Antioxidants can inhibit the oxidation of LDL, thereby reducing the fluorescence intensity. The method was applied to prove that the gelatin-catechin conjugate showed more potent inhibitory activity on LDL oxidation than unconjugated catechin [65].

To better reflect the effects of antioxidants in physiological conditions, the cellular antioxidant activity (CAA) method is created to evaluate the intracellular reaction of antioxidants to establish a better biological correlation with the bioavailability, absorption, and metabolism of antioxidants in cells. The pre-treated cells contained dichlorofluorescin (DCFH) probes. Through the action of 2,2′-azobis (2-amidinopropane) dihydrochloride (ABAP) on cells to generate peroxy radicals, DCFH is oxidized to dichlorofluorescein (DCF) with fluorescence, whose absorption and emission wavelengths are 485 nm and 583 nm respectively. Antioxidants can block the oxidation of DCFH to DCF. Therefore, the antioxidant capacity of antioxidants can be evaluated by reducing cell fluorescence [82]. Fan et al. [17] proved that resveratrol loaded by zein-BSA nanoparticles has higher antioxidant capacity than free state by using the CAA method, indicating that the nanoparticle delivery system improved the absorption and bioavailability of encapsulated antioxidant components into human colon carcinoma cell monolayers (Caco-2 cells). Hoskin et al. [66] found that blueberry polyphenol–protein particles maintained the cellular antioxidant activity of the blueberry extract in mouse macrophage RAW 264.7.

### 3.3. Assessment of Capacities against Lipid Peroxidation

At present, the emulsifier or stabilizer used as a lipid system has become one of the crucial applications of polyphenol–protein complexes. For this purpose, in addition to the above methods for determining antioxidant activity, it is necessary to assess the antioxidant activity of the complex to inhibit the oxidation of the lipid system.

Peroxide is the main primary product of the automatic oxidation of lipids [83]. The peroxide formed by lipid oxidation can oxidize Fe^2+^ to Fe^3+^ under acidic conditions. Then Fe3+ and thiocyanate ions form the red complex, which has the maximum absorption in 480~515 nm. By comparing the peroxide value (POV) of the lipid system with or without antioxidants in a storage time (during the process of lipid oxidation), the effect of antioxidants on inhibiting lipid oxidation can be obtained [84,85]. The second product of lipid oxidation is malondialdehyde (MDA). In the thiobarbituric acid reactive substances (TBARS) method, thiobarbituric acid (TBA) reacts with MDA under acidic conditions to form red compounds with absorption at 532 nm. Thus, the activity of inhibiting lipid oxidation can be evaluated by measuring the amount of MDA via the TBARS method in the lipid system containing antioxidants. By measuring the POV and TBARS value of the emulsion, Wen et al. [26] found that at 6-day, the oxidation degree of ovalbumin emulsion and ovalbumin–procyanidins complexes emulsion was 5.90% and 1.78%, respectively, reflecting that the addition of procyanidins improved the oxidation stability of the emulsion. The results of Zhao et al. [68] showed that the POV and TBARS values of the emulsion added with the conjugate of anchovy protein hydrolysate and phenols (catechin, gallic acid, and tannic acid) decreased significantly during storage time. The ability of the conjugate to inhibit oxidation was consistent with the trend of the antioxidant activity of polyphenols.

In addition, the activity of maintaining the oxidative stability of the lipid system can be evaluated by measuring the number of conjugated dienes (CD) formed by unsaturated fatty acids in the lipid system [86]. Chen et al. [72] evaluated the antioxidant activity of the whey protein isolates-lotus seedpod proanthocyanin conjugate in the emulsion. The results showed that the conjugates had a lower CD value than whey protein isolates alone during the 15-day storage period, reflecting the higher antioxidant stability of the conjugates.

### 3.4. Effects of Assessment Methods on Antioxidant Properties of the Complexes

As described above, various methods exist for evaluating the antioxidant properties of polyphenol–protein complexes. Researchers often use various ways to determine the antioxidant activity of the complexes. Generally, the determination results of different methods are consistent. However, due to the different principles of the methods, sometimes the results are inconsistent. Yin et al. [60] observed that the antioxidant activity of β-casein-chlorogenic acid complexes determined by FRAP and ABTS had opposite results. The results of FRAP showed that the complex has a synergistic effect. That is, the FRAP value of the complex was higher than the sum of β-casein and chlorogenic acid alone. On the contrary, ABTS free radical scavenging ability showed an antagonism effect of the complexes. Another study showed that although the complexes formed by whey protein isolate and EGCG, quercetin, apigenin, or naringenin, all showed higher antioxidant activity than natural whey protein isolate, the antioxidant capacity of the complexes combined with different polyphenols was observed in different orders in the ABTS and DPPH radical scavenging methods and the FRAP method [13]. 

It should be noted that most of the evaluation of the antioxidant activity of the complexes formed by the binding of polyphenols and proteins needs to be compared with natural proteins or phenolic acids alone to show the enhanced antioxidant activity of the complexes [13,61,87] or the masking effect of phenolic acids [11,50,62]. Sometimes, the antioxidant capacity of the complexes will be compared with the sum of the antioxidant activities of individual proteins and phenolic acids to judge the synergistic or antagonistic effect of the forming complexes in terms of antioxidant capacity [60,88,89]. The scholars also pointed out that although pure polyphenols showed more potent antioxidant activity than complexes, if the degree of combination or grafting rate of polyphenols in the complexes were considered in the calculation, it would be found that the combination of proteins and polyphenols synergistically enhanced their antioxidant activity [18].

To sum up, the assessment methods and comparison or calculation methods for evaluating the antioxidant capacity of polyphenol–protein complexes have an essential impact on the results. The choice of methods should take into consideration the experimental purpose or the characteristics of the composite, and the results of the determination should be compared. However, there are relatively few studies on the in vivo antioxidant properties of the complex, which should be given greater attention and more abundant studies in the future to demonstrate better the digestive properties, oral stability, bioaccessibility, and bioavailability of the complexes.

## 4. Factors Affecting the Antioxidant Properties of Polyphenol–Protein Complexes

As mentioned earlier, when preparing the polyphenol–protein complexes, different conditions were applied to obtain the complexes according to various research or application purposes (Figure 3 and Table 1). Therefore, the combination mode involved in the preparation process, the type and concentration of protein or polyphenol selected, and the extrinsic conditions for preparation will all affect the antioxidant properties of the polyphenol–protein complexes (Table 1). 

### 4.1. Combination Mode

Among the interactions between polyphenols and proteins, covalent conjugates and non-covalent complexes usually show different antioxidant activities. The literature reveals that the antioxidant capacity of covalently bound polyphenol–protein conjugates is more potent than that of non-covalent ones in most cases. Xu et al. [48] found that the antioxidant activity of the zein–polyphenol covalent complex was stronger than that of the non-covalent complex, possibly due to the difference in protein secondary structure and hydrophobic group exposure. Gu et al. [38] clarified that covalently bound complexes showed better free radical scavenging activity than non-covalent complexes due to the low grafting rate caused by the removal of polyphenol molecules by dialysis when preparing non-covalent polyphenol–protein complexes. 

In addition, even if polyphenol protein complexes are covalently bound, different preparation methods will also lead to differences in their antioxidant activities. The conjugates prepared by free radical induction have higher antioxidant activity than those prepared by alkaline reaction [19]. Due to the different processes and mechanisms formed by the two paths, the conjugates formed under free radical conditions have more sites available for polyphenol conjugation, resulting in the polyphenol–protein complexes showing better antioxidant capacity [19,38]. 

### 4.2. Polyphenols and Proteins

As the main subjects forming the complexes, polyphenols and proteins are the main factors affecting the antioxidant properties of the complexes. The type or structure of the polyphenols/proteins makes a difference in the binding affinity between them. Moreover, the concentration (ratio) of the two also affects the antioxidant properties of the complexes.

For polyphenols, there is a wide variety of structures, ranging from small individual phenolic acid molecules to polymeric polyphenols. This leads to differences in their binding when interacting with proteins and therefore affects the antioxidant properties of the complexes [4,90]. Liu et al. [13] observed that the antioxidant capacities of whey protein isolate-EGCG conjugates were the most potent, followed by whey protein isolate–quercetin, while the antioxidant capacity of whey protein isolate–apigenin and whey protein isolate-naringenin was the weakest, reflecting that the antioxidant capacity of the conjugates was related to the number of phenolic hydroxyl groups of the polyphenols introduced into the protein. Xu et al. [48] revealed that zein-gallic acid conjugate exhibited more effective free radical scavenging activity than zein-chlorogenic acid conjugate obtained via chemical cross-linking agents (EDC/NHS), reflecting that the number and position of hydroxyl groups on the aromatic ring of polyphenols affected the antioxidant activity of polyphenol–protein complexes. From another point of view, the differences in polyphenol structure, especially the number and position of hydroxyl groups, will lead to different binding modes with proteins, thus affecting the antioxidant activity of the complexes. If the polyphenols and proteins have a strong affinity, the grafting ratio between them will increase [42,90,91]. That is, the polyphenol content introduced in the complexes will increase so that the complexes have higher antioxidant capacity. Xu et al. [42] found that the covalent zein-chlorogenic acid complex obtained by the alkaline reaction exhibited the most potent antioxidant capacity compared with gallic acid and caffeic acid, probably due to the high grafting rate and more hydroxyl groups of the zein-chlorogenic acid complex. However, if the binding with protein involves more hydroxyl groups in polyphenols, the masking effect of the antioxidant activity of the complexes will enhance. For example, the interaction of α-lactalbumin and hydroxy safflower yellow A may involve the most hydroxyl groups. Thus hydroxy safflower yellow A showed the most extensive FRAP loss in the interaction of α-lactalbumin and three similar chalconoids (hydroxy safflower yellow A, neohesperidin dihydrochalcone and naringin dihydrochalcone) compared to pure polyphenols [11]. Stojadinovic et al. [91] also observed a good correlation between the binding affinity of different dietary polyphenols-β-lactoglobulin and the total antioxidant activity of the formed complexes.

A polyphenol–protein complex’s antioxidant capacity is also affected by the structure and type of proteins like that of polyphenols. It has been reported that the ABTS⋅^+^ scavenging ability of the BSA-resveratrol complex is more potent than that of α-lactalbumin–resveratrol and β-lactoglobulin–resveratrol [89]. At the same time, resveratrol and its products may bind to the hydrophobic cavity of BSA, thus providing better protection for the antioxidant capacity of the complexes compared with the other two proteins under irradiation [89]. In addition, different antioxidant activities of α-lactalbumin/β-lactoglobulin/lactoferrin–EGCG complex [92] and whey protein isolate/casein-chlorogenic acid complex [87] were observed, respectively.

The concentration (ratio) of the polyphenols and proteins also affects the antioxidant properties of the complexes. Generally, the higher the concentration of polyphenols, the stronger the antioxidant capacity of the polyphenol–protein complexes formed [52,60,90,93]. Thongzai et al. [52] observed that the antioxidant activity of the whey protein-phenolic complex was affected by the concentration of phenolic acids. In the range of 0.5–5% of phenolic acid concentration, the antioxidant activity of the complexes increased with the increase of phenolic acid concentration. Yin et al. [60] also found that the antioxidant activity of the complexes of β-casein-chlorogenic acid showed an increasing trend in a dose-dependent manner. However, excessive polyphenol concentration would destroy the protein structure, so it harmed the antioxidant properties of the complexes [55,94].

### 4.3. Extrinsic Conditions

#### 4.3.1. Temperature

With the increase in temperature, the binding mode of polyphenols to proteins may change from non-covalent binding to covalent binding, which may lead to changes in the antioxidant capacity of the complexes [60,61,62]. According to the report, the covalent bonding β-lactoglobulin-chlorogenic acid complex was formed at higher temperatures (above 85 °C) and showed better antioxidant activity [61]. However, at a higher heating temperature, the closer combination of polyphenols and proteins would lead to the occupation of active hydroxyl groups in the polyphenol molecules, reducing the antioxidant activity [62]. At the same time, high-temperature lead to the degradation or isomerization of polyphenols, which weakens antioxidant capacities [55,61,95]. 

#### 4.3.2. pH

The binding of polyphenols to proteins can occur at a specific range of pH (4.0–10.0) [96]. As mentioned above, polyphenols are oxidized to quinones under alkaline conditions and then covalently bound to proteins. This leads to changes in the antioxidant activity of the complexes. It has been reported that pH affects the stability of phenolic compounds, the structural characteristics of proteins, and other factors, thus affecting the antioxidant properties of the complex [56]. Wang et al. [95] observed that at pH 8.0, EGCG modified α-lactalbumin covalent complex was formed, and its antioxidant properties were significantly enhanced. In addition, it has been reported that when coupling polyphenols with proteins by carbodiimide crosslinker chemistry method, the highest amount of phenolic acid was introduced to the protein at pH 6, thus obtaining the complex with the best antioxidant activity under this condition [49].

#### 4.3.3. Other Extrinsic Factors

Other extrinsic conditions such as ultrasonic, irradiation, and solvent will affect the antioxidant capacity of polyphenol protein complexes. The changes in protein structure induced by ultrasonic treatment enhanced the affinity between protein and polyphenols. Thus, the more potent antioxidant activity of the complex after ultrasonic treatment was observed [30,50]. The isomerization of polyphenol molecules caused by irradiation also caused changes in the antioxidant activity of polyphenol–protein complexes [89]. Moreover, since the polarity of polyphenols is different, improving the solubility of the complex in different solvents enhanced its free radical scavenging capacity [18].

## 5. Potential Applications of the Polyphenol–Protein Complexes with Antioxidant Properties

**Figure 5:**
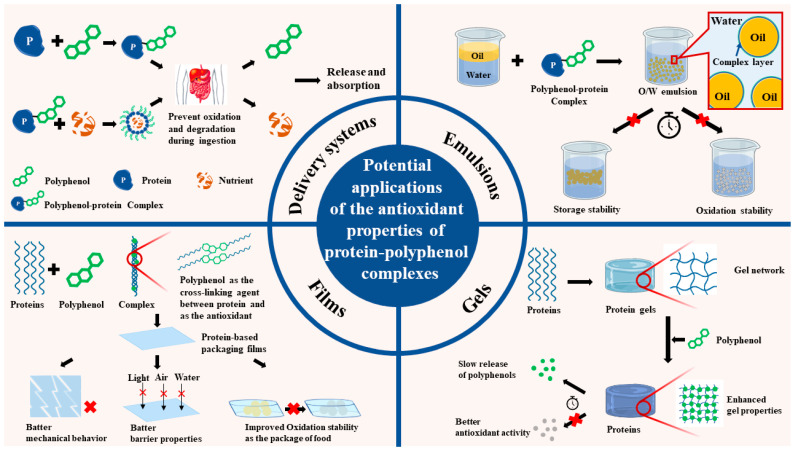
Potential applications of the antioxidant properties of protein–polyphenol complexes.

Complexes with polyphenols and proteins have enhanced their functional properties and antioxidation activities. The complexes may be used for delivery systems, emulsions, protein-based films, and gels. (Figure 5).

### 5.1. Delivery Systems

The amphiphilic nature of proteins is widely considered an excellent carrier for bioactive compounds, especially for improving the bioaccessibility and stability of some poorly soluble polyphenols (e.g., curcumin, resveratrol, quercetin) [14,97,98]. When delivering polyphenols, polyphenols will form complexes with the protein carriers and enter the digestive system. At this time, the formation of the complexes helps to protect polyphenols from degradation so that the antioxidant activity of polyphenols and other biological activities still exist when they reach the intestinal tract [3,99]. Tong et al. [50] confirmed that the antioxidant activity of protein fibril and EGCG complex was significantly increased, and EGCG could be prevented from degradation under the protection and slow-release function of the protein, so it had higher biological accessibility. Jiang et al. [87] observed that the digested products of the complex have the effect of synergetic scavenging free radical capacity through the simulated gastrointestinal digestion experiment. The quercetin and resveratrol delivered by zein–carboxymethyl cellulose nanoparticles had enhanced oxidation resistance and storage stability [100]. Meanwhile, some protein delivery systems for polyphenols have been developed. For example, as a low-cost, safe, and effective carrier, zein nanoparticles improved the oral bioavailability of resveratrol [101] and helped to retain the antioxidant activity of resveratrol [102]. Alternatively, composite carriers formed by combining proteins and polyphenols are also highly promising as carriers for active substances. Zein cross-linked EGCG nanoparticles were developed for the co-delivery of curcumin and resveratrol. The delivery system showed better stability and improved biological accessibility for the delivered nutrients due to its antioxidant and encapsulation effects [103]. Similarly, since BSA-caffeic acid covalently bound nanoparticles had higher antioxidant activity than BSA alone, resveratrol in the composite nanoparticles showed better heat and ultraviolet resistance stability and had better cell antioxidant activity [17]. Therefore, the orientation of polyphenol–protein complexes as delivery systems has essential application value in medicine and in vivo therapy.

### 5.2. Emulsions

The excellent emulsifying and antioxidant properties of polyphenol–protein complexes have been observed and applied to food emulsion systems [6]. The proteins modified by polyphenols show higher emulsifying activity and emulsion stability due to the increase in surface hydrophobicity and the decrease in particle size [6]. Fei et al. [104] found that the thermal stability, antioxidant capacity, solubility, and emulsion stability of whey proteins were improved after cross-linked with gallic acid/protocatechuic acid through covalent bonding. Furthermore, polyphenols significantly improve the oxidation resistance of the emulsions, which improves their antioxidation and storage stability [7]. Wang et al. [105] showed that 0.03% EGCG-modified chicken wooden breast myofibrillar protein retarded phase separation by preventing droplet and protein aggregation, significantly reduced the particle size of the emulsion, and improved emulsion stability and emulsion activity. Also, the inhibition of protein oxidation and lipid oxidation by 0.03% EGCG was recorded during storage at 50 °C for 96 h. 

**Figure 6:**
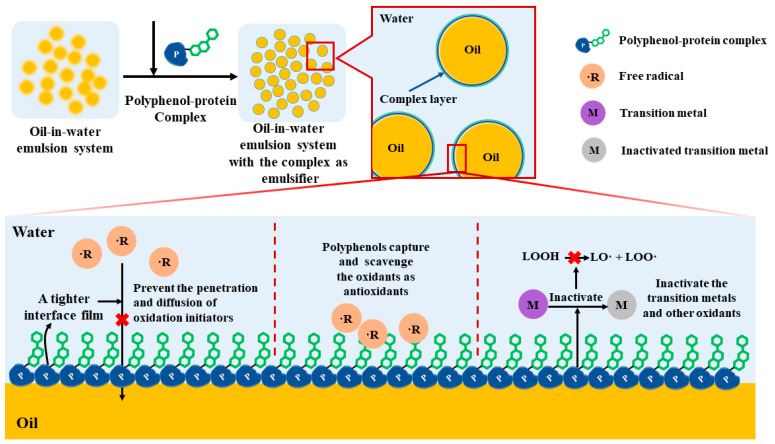
The mechanism of polyphenol–protein complex synergistically enhancing the oxidation stability of oil-in-water emulsions system.

Lipid oxidation has been a pressing difficulty for oil–water emulsion systems in food applications. The contact area between lipids and water is the crucial area for the development of oxidation. The complexes can form a tighter interface facial mask in this area as the result of the decrease of the interfacial tension after the conjugation of the proteins and polyphenols, which can effectively prevent the penetration and diffusion of oxidation initiators. At the same time, the potent antioxidant property of polyphenols can capture the oxidants at this interface to avoid lipid oxidation [6]. In addition, the complexes also inactivate the transition metals and other oxidants at the oil–water interface, thus preventing the decomposition of lipid hydroperoxide (LOOH) into alkoxy (LO·) and peroxy (LOO·) radicals [6,43] (Figure 6). Based on this, polyphenol-modified protein complexes were used to develop emulsifiers with anti-lipid oxidation properties. The combination of whey protein isolate and lotus seedpod proanthocyanin significantly enhanced antioxidant activity, and the conjugated diene production of flaxseed oil encapsulated by the complex is lower, showing better antioxidant stability [72]. Studies have shown that anchovy protein hydrolysate–polyphenol (catechin, gallic acid, tannic acid) conjugates effectively improved the physical stability and oxidative stability of fish oil emulsion during storage [68]. The reduced thiobarbituric acid reactive substances and peroxide value proved that EGCG-conjugated egg albumen hydrolysate improved the oxidative stability of fish oil emulsion [106]. Similarly, the enhanced emulsification ability, the free radical scavenging activity, and the antioxidant effect of linoleic acid oxidation exhibited by the oyster water-soluble protein–EGCG conjugate during storage in the linolic acid emulsion system suggested that the conjugate possessed both emulsification and antioxidant abilities [107]. Moreover, the chickpea protein isolate combined with gallic acid by alkali treatment contributed to the accumulation of higher gallic acid at the droplet interface, and the reduction of primary and secondary oxidation products reflected the chemical stability of the emulsion [108]. It was reported that the soybean protein 7S/11S-rutin covalent conjugates formed an interfacial barrier and exhibited excellent free radical scavenging properties while inhibiting lipid oxidation in the algae oil-enriched emulsions during storage [109]. 

### 5.3. Films

Due to the characteristics of environment-friendly and degradable regeneration, proteins have become a good source of food packaging films [110]. Polyphenols can not only be used as a cross-linking agent between protein molecules to make up for the poor mechanical and barrier properties of protein-based films but also as an antioxidant to enhance the antioxidant capacity of protein-based films [20,111]. The incorporation of ferulic acid was reported to result in denser film microstructure, lower water vapor permeability, and significantly improved antioxidant activity of the gelatin nanocomposite packaging films [112]. It was observed that the high antioxidant properties of fish gelatin-based film incorporated with mangrove extracts (rich in polyphenols) were favorable to inhibit oxidation and showed first potential as active packaging materials [113]. More importantly, the ability of polyphenol-grafted protein-based films to improve the storage stability of foods has also been demonstrated. Nilsuwan et al. [114] found that chicken protein isolate/fish gelatin blend film added with gallic acid or tannic acid had enhanced mechanical properties. The film containing 0.75% gallic acid had high ultraviolet-light barrier properties and antioxidant activity. The oxidation of chicken skin oil sealed with the forming pouches was delayed. Similarly, a fish gelatin–EGCG composite film was developed for packaging pouches of chicken skin oil [115]. The results showed that the chicken skin oil in the pouches showed a low oxidation level, and EGCG added to the gelatin pouches could retain the unsaturated fatty acids in chicken skin oil [115]. A new type of fish glue film incorporated with protocatechuic acid was developed, and it was found that it had improved flexibility, ultraviolet, and water resistance. In terms of food preservation, the composite film has antioxidant and antibacterial activities, which improves the security of beef preservation and extends the shelf-life [116]. 

### 5.4. Gels

Proteins can form gel networks triggered by certain environmental conditions (temperature, pressure, pH, mineral antiparticles, crosslinkers, etc.) [40]. Polyphenols added as protein cross-linking agents can enhance the gel properties of proteins [7]. Man et al. [117] reported that samples of 0.002 and 0.01 g/g CA-modified soy protein isolate had significantly enhanced gel strength due to greater crosslink density, and the covalent interactions facilitated the development of the gel network. Xue et al. [118] also reported that the water-soluble polymer of tea polyphenol–egg white had improved gel strength under thermal treatment, and therefore, tea polyphenol could be used as an excellent gel modifier in egg white products. In addition, the protein conjugated with polyphenols imparted antioxidant properties to the gel, expanding its functional scope and application scenarios. For example, a gel of gelatin covalently modified with gallic acid exhibited enhanced antioxidant activity and antimicrobial activity, demonstrating its potential for relevant food and medical applications [119]. Furthermore, proteins can also play a role in the controlled release of polyphenols [120]. More importantly, the combination of the two can better play the role of antioxidation under the slow release of polyphenols created by the enhanced gel characteristics [4]. The mixture of catechin and gelatin has the potential to become an effective antioxidant biomaterial since its enhanced antioxidant activity and slow release of catechin obtained from more robust gel properties have been observed [121]. Polyphenol-containing gelatin/chitosan hydrogels cross-linked by laccase have shown that their structural stability is enhanced by the polyphenols. In particular, the composite hydrogel had better antioxidant activity and inhibition of chronic wound enzymes in biological activity [122].

## 6. Conclusions and Perspectives

Generally, polyphenols and proteins interact to change their functional properties. The improvement of antioxidant capacity is one of the most concerning aspects. The combination of the two can be formed by covalent or non-covalent methods. The antioxidant activity of the complexes can be assessed by measuring their ability to scavenge free radicals or their ability to protect lipids from oxidative damage. Due to the type of protein and polyphenol, binding mode, determination method, environmental conditions, and other factors, differences in the antioxidant properties of the complexes have been observed. Improved antioxidant activity can be achieved using polyphenols combined with proteins applied in emulsions, films, gels, and active substance delivery systems. However, the necessary cellular and animal experiments should be more widely used to provide evidence for the biological activity, more importantly, toxicity and safety of the complexes. More types and sources of proteins and polyphenols for forming complexes should also be studied to expand their applications in food engineering.

## Figures and Tables

**Table 1:** Measurement methods and changes in antioxidant activity of polyphenol–protein complex.

Measurement Methods	Proteins	Polyphenols	Reaction Conditions	Influence on Antioxidant Activity of Polyphenol–Protein Complexes	Mechanism Explanations	Ref.
DPPH method ABTS method	β-lactoglobulin	Ferulic acid	pH 9.0 at 20 °C for 24 h	The free radical scavenging ability of the complexes was significantly improved	The introduction of hydroxyl groups was the reason for the enhanced antioxidant activity of the complexes	[51]
	Zein	Chlorogenic acid, gallic acid, and caffeic acid	pH 12.0 at 25 °C under atmospheric air for 24 h	The antioxidant activity changes of control zein and zein–polyphenol complexes	The synergistic antioxidant effect of polyphenols	[42]
	Zein	Chlorogenic acid and gallic acid	pH 6.5 cross-linked by EDC and NHS	The antioxidant activity of zein was enhanced by covalent grafting of polyphenols	The hydroxyl groups in polyphenols were still available after conjugation and terminate the radical chain reaction	[48]
	Whey protein	Gallic acid, ferulic acid, and tannic acid	pH 9.0 at 25 °C under atmospheric air for 24 h	The phenolic compounds contributed to the increase in the antioxidative activity of the modified whey protein	The antioxidative activity of the complex is dependent on the type and concentration of phenolic compounds	[52]
	β-lactoglobulin	Chlorogenic acid	25 °C for 24 h	The addition of chlorogenic acid improved the free radical scavenging ability of β-lactoglobulin	The protein combined with polyphenols showed better antioxidant capacity	[30]
	β-lactoglobulin	Cyanidin-3-O-glucoside(C3G)	pH 9.0 at 20 °C for 2 h	The free radical scavenging capacities of complexes were improved	C3G conjugation imparted excellent antioxidant properties to the complexes	[53]
	Wheat gluten hydrolysate	Chlorogenic acid	H_2_O_2_/ascorbic acid, under atmospheric air at 25 °C for 2 h	The radical-scavenging activity of conjugates increased significantly	The structure changed, and consequently, some antioxidant amino acids exposed after forming conjugates	[46]
	Camel whey	Quercetin	H_2_O_2_/ascorbic acid, under atmospheric air at 25 °C for 24 h	The radical scavenging activity of the conjugates produced was enhanced	The quercetin contributed OH groups after conjugation	[47]
	Protein isolate from large yellow croaker roe	EGCG	H_2_O_2_/ascorbic acid, under atmospheric air at 25 °C for 24 h	the conjugation significantly increases the antioxidant capacity of native protein	The better antioxidant capacity of the conjugate may be a result of the addition of EGCG to increase the hydroxyl content	[54]
ORAC method	Mung bean globulin	Mung bean polyphenol	At 25, 70, 85 or 100 °C for 2 h	With increased addition of polyphenols, the antioxidant capacity of the system showed a trend of first increasing and then decreasing slightly	The combination of polyphenols with globulins led to the introduction of phenolic hydroxyl groups that can scavenge free radicals. When the interaction ratio was larger, the sites that could supply hydrogen and electrons to free radicals were masked	[55]
	Whey protein	Caffeic acid and EGCG	pH 3.5 or 7.0 at 25 °C for 60 min in the dark	The complexation suppressed the antioxidant capacity compared to the isolated compounds	The suppression may be due to hydrophobic interaction and H-bonding between these compounds	[56]
	Ovalbumin	Catechin	H_2_O_2_/ascorbic acid, under atmospheric air at 25 °C for 24 h	The antioxidation ability of ovalbumin was improved via its conjugation with catechins	The conjugation introduced a large amount of phenolic hydroxyl groups	[43]
	Whey protein	EGCG	H_2_O_2_/ascorbic acid, under atmospheric air at 25 °C for 24 h	The conjugate exhibited stronger antioxidation ability than whey protein	The presence of EGCG resulted in the increase of hydroxyl groups in whey protein	[57]
HRSA method	β-lactoglobulin	Caffeic acid	pH 2.5, 6.0, or 8.5, cross-linked by EDC and NHS	The activity of the complex significantly higher than the un-derivatized β-lactoglobulin	-	[49]
	β-lactoglobulin	Curcumin	pH 6.0 or 7.0 at 25 °C	In the presence of β-lactoglobulin, the antioxidant capability of complexes is remarkably higher than curcumin alone	The high activity of complexes may be contributed by both curcumin and β-lactoglobulin	[58]
	Myofibrillar protein	Hydrophilic and hydrophobic	pH 9.0 at 25 °C	The incorporation of polyphenol enhanced the antioxidation activities	The enhanced antioxidation activities were related to the hydroxyl groups substituents in a polyphenol ring	[59]
FRAP method	β-casein	Chlorogenic acid	pH 7.0, at 25 or 65 °C for 30 min, 100 or 121°C for 15min	Complexes showed a synergetic effect on FRAP activity	The reducing groups originally buried in β-casein are exposed and enhance the FRAP of the complexes	[60]
	β-lactoglobulin	Chlorogenic acid	pH 7.0, at 25, 65 or 85 °C for 30 min, 100 for 15min, or 121°C for 10 min	The addition of β- lactoglobulin could enhance chlorogenic acid′s ability to resist thermal oxidation	The complexes protected the chlorogenic acid from oxidation reaction	[61]
	β-lactoglobulin	EGCG	pH 7.0, at 25, 65 or 85 °C for 30 min, 100 for 15min, or 121°C for 10 min	The addition of β- lactoglobulin inhibits the antioxidation ability of EGCG	The formation of complex leads to the occupation of active hydroxyl group in EGCG	[62]
	Whey protein isolation	EGCG, quercetin, apigenin, and naringenin	H_2_O_2_/ascorbic acid, under atmospheric air at 25 °C for 24 h	The complexes showed higher antioxidant activity, especially whey protein isolation-EGCG complex	A large number of phenolic hydroxyl groups were introduced into whey protein isolation	[13]
	Lentil protein	Quercetin, rutin and ellagic acid	pH 9.0, at 25 °C under atmospheric air for 24 h	The combination of polyphenols and proteins synergistically improves their antioxidant capacity	Coupling of polyphenols to lentil protein imparted protein reduction ability	[18]
	α-lactalbumin	Hydroxy safflower yellow A, neohesperidin dihydrochalcone and naringin dihydrochalcone	pH 7.0, at 25 °C	FRAP of the complex is significantly lower than that of its corresponding phenolic acid alone	The hydrogen bond between α-lactalbumin and chalcone is formed through hydroxyl, thus occupying hydroxyl	[11]
Metal-Chelating method	Porcine plasma protein hydrolysates	Tannic acid and oxidized chlorogenic acid	pH 9.0, at 25 °C under atmospheric air for 24 h	Improved metal chelating activity by trapping transition metals	The incorporation of phenolic compounds improves the antioxidant activity	[63]
	Silk sericin	Hydroquinone and pyrogallol	pH 9.0, at 25 °C under atmospheric air for 24 h	The metal chelating activity of the conjugates was improved	The hydroxyl groups of phenolic compounds can quench oxidants by providing hydrogen atoms	[64]
·O^2−^ scavenging activityLDL oxidation	Gelatin	Catechin	Laccase, pH 7.0, at 20 °C for 24 h, under atmospheric air	Conferred the SOD-like antioxidant activity on gelatin, improved antioxidant activity of inhibiting oxidation of LDL	-	[65]
CAA method	Zein and bovine serum albumin	Resveratrol and caffeic acid	H_2_O_2_/ascorbic acid, under atmospheric air at 25 °C for 24 h	Resveratrol-loaded complexes exhibited higher antioxidant ability than free resveratrol	The complex nanoparticles improved chemical stability of the delivery system	[17]
	wheat protein, chickpea protein and soy protein isolate	Blueberry polyphenol	Protein-rich substrates were added to blueberry extract, and then spray drying or freeze-drying	Improved cellular antioxidant activity	-	[66]
	Wheat gluten hydrolysate	Chlorogenic acid	H_2_O_2_/ascorbic acid, under atmospheric air at 25 °C for 2 h	The conjugates showed significantly synergistically increased effect cellular antioxidant activity	The covalent binding enhanced the ability to promote the entry of chlorogenic acid into cells	[46]
	Wheat gluten hydrolysate	Chlorogenic acid	Interaction during in vitro digestion	The CAA of the mixture was higher than that of chlorogenic acid or hydrolysate alone	The interaction enhanced the cell entry and the stability of chlorogenic acid	[67]
POV methodTBARS method	Anchovy protein hydrolysate	Catechin, gallic acid and tannic acid	pH 9.0, at 25 °C under atmospheric air for 24 h	The POV level exhibited a remarkable reduction with the addition of conjugates	The conjugates could serve as the electron or hydrogen atom donors, leading to the break of free radical chain and reacting with certain peroxide precursors to prevent the formation of peroxides	[68]
	Ovalbumin	Procyanidin	pH 7.4, at 25 °C for 1 h	The oxidation degree of ovalbumin– procyanidin emulsion was lower than that of ovalbumin emulsion	The interaction altered the sensitivity of oxidation on ovalbumin and improved the ability to scavenge free radicals	[26]
	Soy protein isolate	EGCG	pH 9.0, at 4 °C under atmospheric air for 24 h	the Emulsions stabilized by the complexes exhibited better antioxidant capacity	EGCG delayed oil oxidation by donating a hydrogen from the hydroxyl groups and reduce the reactivity of the transition metal ions and oil by metal chelation	[69]
	Soy protein isolate	EGCG	pH 9.0, at 4 °C under atmospheric air for 24 h	The complexes provided superior oxidation resistance compared to pure protein	The proteins adsorbed at the oil–water interface is more sensitive to oxidation than unabsorbed proteins	[70]
	Oleosin	EGCG	pH 9.0, at 25 °C under atmospheric air for 12 h	The emulsion with the complexes shows high oxidative stability	the complexes had the ability to scavenge free radicals and chelate metal ions	[71]
CD method	Whey protein isolates	lotus seedpod proanthocyanin	H_2_O_2_/ascorbic acid, under atmospheric air at 25 °C for 24 h	The conjugate exhibited stronger antioxidant effects than then WPI alone	—	[72]
	Pea protein	Tannic acid	pH 7.0, at 25 °C under atmospheric air for 30 min	The lipid oxidation rate decreased with increasing tannic acid concentration in the emulsions	Tannic acid endowed the complexes with antioxidant activity and led to the formation of a thicker and denser coating around the oil droplets	[73]

DPPH: 2,2-diphenyl-1-picrylhydrazyl radical; ABTS: 2,2′-azinobis (3-ethylbenzthiazoline-6-sulfonic acid) radical; ORAC: Oxygen radical absorbance capacity; HRSA: Hydroxyl radical scavenging activity; FRAP: Ferric ion reducing antioxidant power; ·O^2−^: Superoxide anion radical; LDL: Low-density lipoprotein; CAA: Cellular antioxidant activity; POV: Peroxide value; TBARS: Thiobarbituric acid reactive substances; CD: Conjugated dienes; EGCG: (−)-Epigallocatechin-3-gallate; EDC: 1-ethyl-3-(3-dimethylaminopropyl) carbodiimide hydrochloride; NHS: *N*-hydroxysuccinimide.
